# Is Bariatric Surgery Safe in Cirrhotics?

**DOI:** 10.5812/hepatmon.8536

**Published:** 2013-02-25

**Authors:** Roger Wu, Jorge Ortiz, Ramsey Dallal

**Affiliations:** 1 Albert Einstein Medical Center, Philadelphia, USA

**Keywords:** Obesity, Liver Cirrhosis, Bariatric Surgery, Anastomosis, Roux-en-Y, Gastrectomy

Obesity is associated with an increase in mortality and primary graft nonfunction after liver transplantation (LT) (1). Weight loss is recommended for obese patients in need of LT. Diet, exercise, or medications are rarely successful. Conversely, bariatric surgery can allow patients to achieve significant and sustained weight loss, leading to improvement of obesity-associated comorbidities such as hyperlipidemia and diabetes (2-4).

Bariatric surgery may be useful in cirrhotics needing LT who were denied evaluation primarily because of weight. We examined the medical literature concerning the safety and efficacy of bariatric surgery in cirrhotics by conducting a literature search using MD Consult, Cochrane, Ovid, and Medline with keywords “cirrhosis,” “bariatric,” and “obesity surgery.” Studies in English through January 2012 were included. We recorded information about demographics, type of bariatric surgery, and surgical outcome.

Three articles were identified, giving a combined total of 44 patients with cirrhosis undergoing bariatric surgery (5-7). Laparoscopic surgery was performed in two studies (Dallal et al., Takata et al.). The third (Brolin et al) employed open approaches (Table). The mean age at surgery was 49.5 years. The average BMI before surgery was 52.5 kg/m^2^. Where reported, all patients were Child class A or B. Most of the patients (32, 73%) were found to have cirrhosis unexpectedly during surgery.

**Table:** Demographics, Surgical Information, and Complications

**No. of Patients**	44
**Male patients, No. (%)**	15 (34)
**Mean age at time of surgery, y**	49.5
**Mean BMI before surgery**	52
**Type of surgery performed**	
Laparoscopic Roux-en-Y	27
Open Roux-en-Y	7
Laparoscopic sleeve gastrectomy a	9
Ileojejunal bypass	1
**Intraoperative deaths**	0
**Perioperative deaths**	1
**Average estimated blood loss, mL **	304
**Complications, %**	14 (32)
Acute tubular necrosis	4
Significant blood loss	3
Prolonged intubation	2
Fluid leak	2
Encephalopathy	1
Prolonged ileus	1
Marginal ulcer	1

^a^Three additional patients underwent sleeve gastrectomy but were excluded from further analysis in one paper

27 patients underwent laparoscopic Roux-en-Y gastric bypass (RYGB), seven patients underwent open RYGB, 9 patients underwent laparoscopic sleeve gastrectomy, and 1 patient underwent jejunoileal bypass. 3 patients who underwent laparoscopic banding were excluded from one of the studies. 21 patients (48%) were followed at least 9 months with an average percentage of excess weight loss (EWL) of 54.2%.

Dallal et al. reported that the mean operative time was 4 hours, while Takata et al. reported a mean operative time of 2.4 hours. Operative time was not mentioned in Brolin et al. There were no intraoperative deaths; one patient died during the perioperative period from acute hepatic decompensation with hepatorenal syndrome. Postoperative complications occurred in 14 patients (32%) (Table).

Dallal et al. noted that the average estimated blood loss was over twice that of patients without cirrhosis. However, a mean estimated blood loss of 58 mL was reported for the 6 patients in Takata et al. Estimated blood loss in the open approach (Brolin et al.) was generally 150-300 mL.

There was considerable heterogeneity in terms of type of surgery performed and the reporting of demographics and outcomes. However, the data suggest that bariatric surgery can be successfully performed in carefully selected patients with cirrhosis, resulting in substantial weight loss, although the risk of certain complications such as renal failure and blood loss may be somewhat higher. Blood loss appears to be less with a laparoscopic approach.

Recently published guidelines on the management of nonalcoholic fatty liver disease (NAFLD) do not make any recommendations regarding the utility of bariatric surgery on obese patients with cirrhosis due to NAFLD (8). Currently, there is no consensus on what bariatric modality is best for a patient with cirrhosis. It does appear that less invasive, laparoscopic procedures are safer to perform. Currently, the three most widely used laparoscopic modalities are RYGB, sleeve gastrectomy, and gastric banding, each having distinct advantages and disadvantages. RYGB presents unique challenges for a patient with cirrhosis, as endoscopic access to the stomach remnant is rendered impossible; this is of particular concern in the setting of a GI bleed or biliary obstruction. In addition, RYGB is often complicated by malabsorption and vitamin deficiencies. Gastric banding appears to be the least invasive of the three laparoscopic modalities. It allows for an effective degree of weight loss, but there is a significant potential risk of infection with the implementation of a foreign device, particularly in the setting of ascites. The FDA currently lists cirrhosis, portal hypertension, and other conditions predisposing to upper gastrointestinal bleeding, such as varices, as contraindications to placement of a gastric band (9). Finally, laparoscopic sleeve gastrectomy, which results in a significant reduction in stomach volume, does not cause malabsorption and does not require implantation of a foreign body. However, there is the risk for significant bleeding in a patient with gastric varices as a portion of the stomach is removed.

It remains unknown whether certain bariatric surgery modalities may complicate future orthotopic liver transplantation. Of the three modalities, RYGB has the most potential to affect future LT. Given the lack of literature comparing the safety and long-term outcomes, the decision of what modality to undergo should be individualized to the patient’s known medical comorbidities. Although there are significant risks with any of these surgical procedures in a cirrhotic, the potential benefits of weight loss and transplant candidacy may outweigh the risks, and it may be reasonable to proceed with bariatric surgery after careful discussion with the patient. While successful bariatric procedures have been demonstrated in patients with relatively preserved hepatic function, as implied from the Child class, it remains unknown whether such surgery may be performed successfully in patients with a higher Child class or MELD score.

We do not know how many patients with cirrhosis were excluded from surgery because of advanced, decompensated cirrhosis. Additionally, the decision was made to abort surgery in some patients who were found to have cirrhosis intraoperatively. There are insufficient data to assess whether complications are higher when a certain surgical modality is used. MELD scores and portal pressures were not calculated in any of the studies. Post-operative weight loss was only reported for 21 of the 44 patients in this study. Child class was reported in only 2 of the 3 papers. Long-term outcomes are not available. It is unknown whether any of these patients underwent LT after surgery.

Given the paucity of data available, we suggest that a registry be created, perhaps under the United Network for Organ Sharing (UNOS). This registry would record the outcomes, complications, and transplant status of obese cirrhotics undergoing bariatric surgery. Such a database would significantly improve our knowledge of the relationship between bariatric surgery and cirrhosis and help establish guidelines where such a surgery may be safely performed and beneficial.
